# Modified *os sepiae* of *Sepiella inermis* as a low cost, sustainable, bio-based adsorbent for the effective remediation of boron from aqueous solution

**DOI:** 10.1007/s11356-022-20578-3

**Published:** 2022-05-20

**Authors:** Sneha Bhagyaraj, Mohammad A. Al-Ghouti, Mariam Khan, Peter Kasak, Igor Krupa

**Affiliations:** 1 Center for Advanced Materials, Qatar University, P.O. Box 2713, Doha, Qatar; 2 Environmental Science Program, Department of Biological and Environmental Sciences, College of Arts and Sciences, Qatar University, P.O. Box: 2713, Doha, Qatar

**Keywords:** *Os sepiae*, Boron, Adsorption, Upcycling, Water treatment

## Abstract

The occurrence of boron in low concentration is essential; however, a higher concentration of boron source in water has a toxic effect on humans as well as have retard effect on agricultural plant growth. Thus, the affordable and facile method to remediate water from higher boron concentrations is highly demanded. This report explores the ability of naturally occurring sustainable bio-waste *os sepiae* (cuttlefish bone, CFB) as an effective adsorbent for the removal of boron from water. Chemical activation of the *os sepiae* powder was examined to improve the efficiency of boron adsorption. A batch adsorption study for boron considering various parameters such as chemical modification of *os sepiae*, pH, initial boron concentration, and the temperature was scrutinized. Untreated (CFB), alkali-treated (CFB-D) and acid-treated (CFB-A) *os sepiae* powders were investigated and the adsorption capacities reached up to 53.8 ± 0.04 mg/g, 66.4 ± 0.02 mg/g and 69.8 ± 0.02 mg/g, respectively, at optimal pH 8 and 25 °C. Boron adsorption by CFB, CFB-D, and CFB-A were well fitted with the linear Freundlich adsorption isotherm model with a correlation coefficient of 99.4%, 99.8%, and 99.7% respectively. Thermodynamic parameters indicated that the adsorption of boron by CFB is an exothermic process and more feasible at a lower temperature around 25 °C. Moreover, detailed morphological and chemical characterization of the influence of adsorbed boron on adsorbents was conducted and discussed. The Fourier transform infrared spectroscopy (FTIR) and X-ray photoelectron spectroscopy (XPS) analysis spectra confirms the involvement of various functional groups including amino, carbonate (CO_3_)^2−^, and hydroxyl groups on the adsorbent in the adsorption mechanisms for boron removal. The results indicate that CFB can be an excellent example for the recycling and reuse of biowaste for water remediation.

## Introduction

The drastic change in climate and the population growth around the world have resulted in the dwindling of drinking water in many parts of the globe. The Middle East countries, which lack annual rainfall to refill their water source and their geological features like arid and semi-arid regions, greatly depend on desalinated seawater for drinking and agricultural purposes (Mazzoni and Zaccagni 2019). For desalination purposes, thermal and membrane-based techniques are mainly employed (Ahmed et al. 2021; Tu et al. 2010). However, membrane-based desalination techniques are not efficient in removing all the trace elements like boron existing in the form of boric acid or borate, which can easily pass through the membrane (Tang et al. 2017). Therefore, researchers around the globe are looking for an alternative, eco-friendly, and economic technologies, which can remove the trace elements from water to bring down their concentration to an acceptable level. Various adsorbents derived from eco-friendly sources have been systematically studied to remove pollutants like metals (Karmacharya et al. 2016), dyes (Stavrinou et al.^2020^), and organic compounds (Turk et al. 2022) from water.

Boron in low concentration is an essential element, which is naturally present in the environment in the form of boric acid and borates by combining with oxygen and other elements (Holleman and Wiberg 2001). In water, boron occurs in the form of boric acid (B(OH)_3_) and borate ${(B(OH)}_{4}^{-})$, and the concentration of boron in seawater achieves approximately 5 mg/L (Hilal et al. 2015). However, consumption of boron above permissible levels by humans, animals and plants will result in various health hazards as a result of boron toxicity (Öztürk et al. 2008). Excess boron causes various adverse effects like nausea, fatigue, and headache in humans (Hadrup et al. 2021) and it retards plant growth (García-Sánchez et al. 2020). As per updated World Health Organization (WHO) guidelines, the permissible level of boron in drinking water is proposed to be 2.4 mg/L (WHO guidelines 2011), however, the limit in the EU (Weinthal et al. 2005) or Middle East countries is down to 1 mg/L (Abdul Rahman 2014).

Unlike other essential elements in the periodic table, boron is one of the elements, which is problematic and challenging in water purification technology. Except for thermal desalination, there is no effective method for the elimination of boron from water. Various methods have been studied for boron removal including reverse osmosis (Li et al. 2020), chemical precipitation (Lin et al. 2022), adsorption (Demey et al. 2014), membrane bioreactors (MBR) (Darwish et al. 2017), and electrodialysis (Noguchi et al. 2018). Among them, the adsorption method attracted the attention of researchers due to its simple operating conditions, economic feasibility, and ability to be applied for water treatment at low concentrations (Bhagyaraj et al. 2021). Several adsorbents have been studied for the removal of boron from water including biopolymers (Ruiz et al. 2013), activated carbons (Melliti et al. 2020; Foo et al. 2013), modified membranes (Çermikli et al., 2020), modified industrial (Babiker et al. 2019) and biowastes (Al-Ghouti and Khan 2018), modified silica-polymer composites (Li et al. 2011), clay and clay nanocomposites (Karahan et al. 2006; Cengeloglu et al. 2007) and so on.

*Sepiella inermis* (Cuttlefish) is a marine animal with a size range of 15 to 25 cm in which the internal bone size occupies a major portion (Chakraborty and Joy 2017). Os sepiae (Cuttlefish bone, CFB) is hard, brittle, and has a layered structure with high porosity and permeability. The main constituents of CFB include biogenic calcium carbonate (CaCO_3_) (89–94%), β-chitin (3–4%), and protein (3–7%) (Chakraborty et al. 2016). Natural CaCO_3_, which exists in the form of orthorhombic aragonite crystal structure, has the ability to adsorb heavy metals from aqueous solutions (Du et al. 2011). Ben Nasr et al. reported the efficacy of cuttlefish bone to remove the fluoride from water as 80% at pH 7.2 at 15 g/L adsorbent dose and 5 mg/L initial fluoride concentration (Nasr et al. 2011). In another study, cuttlefish bone modified using carbonization at 400 °C was used to remove Pb (II) from water. The optimum condition for Pb (II) removal was at pH 4 with 0.2 g/L of the adsorbent dose (Vibhatabandhu and Srithongouthai 2017). Li et al. 2010 reported the use of *os sepiae* as a promising adsorbent for metal removal including Cu, Fe, Zn, and Cd from electroplating wastewater (Li et al. 2010).

The aim of this work is to evaluate the efficacy of *os sepiae* as neat powder and after different modifications on the adsorptive removal of boron from an aqueous medium. *os sepiae*, a bio-waste is low cost, environmentally friendly, and easy processable which attracts its possible application on a large scale. To the best of our knowledge, so far these materials have not been studied for the remediation of boron from water. Various factors affecting the adsorption process such as pH, initial concentration of boron, and temperature were investigated. Moreover, the interaction between the boron species and the adsorbents is discussed and various theoretical models, spectral and microscopic techniques have been analyzed to understand the boron adsorption mechanism.

## Materials and methods

### Materials

The adsorbent used was processed *os sepiae* of Sepiella inermis, which was obtained from the seashore of Al Wakra, Qatar. Anhydrous boric acid (H_3_BO_3_), hydrochloric acid, nitric acid, ethylenediaminetetraacetic acid (EDTA), and sodium hydroxide were obtained from Merck, Darmstadt, Germany. All chemicals were used as such. Ultra pure water (prepared by Purification System Direct Q3, Millipore Corporation, Molsheim, France) was used to prepare all solutions.

### Preparation of unmodified sample (CFB)

The collected bones were washed thoroughly with double distilled water to remove all dirt and contaminants like sand. The sticky organic waste from the surface was removed using scratching. The cleaned *os sepiae* was immersed in boiling water at 100 °C for 30 min to remove all adsorbed impurities, dried at 110 °C for 24 h, and allowed to cool in a desiccator. The cooled CFB was crushed and sieved to obtain particles with a size range of 150–200 µm.

#### Modification using NaOH (CFB-D)

Alkali treatment of the CFB powder was done by treating the powder with 1 N NaOH at 100 °C for 60 min. After treatment, the CFB power is filtered and washed many times with distilled water to remove excess NaOH. Finally, the washed CFB powder is dried at 100 °C for 24 h, allowed to cool in a desiccator.

#### Modification using EDTA (CFB-A)

Activated cuttlefish bone was prepared using treatment with EDTA. Initially, CFB is cut into small pieces and immersed in boiled water for 30 min to remove dirt, and then dried at 100^o ^C for 24 h. The cleaned bones were soaked in 0.03 N HCl for 20 min to create an extended microporous system and washed with distilled water until pH reached 7. The treated bones were filtered, dried, and immersed in 0.25% (w/v) EDTA solution for 20 min to barely decalcify the structure of CFB. The produced bones were finally dried at 100 °C for 24 h, allowed to cool in a desiccator, crushed, and sieved to fine grains of 150–300 µm in size.

### Characterization of the adsorbents

The functional groups present in the adsorbents were analyzed using a Fourier transform infrared (FTIR) spectroscopy technique using PerkinElmer Spectrum 400 spectrophotometer (Waltham, MA, USA). The scanning was done in the range 500–4000 cm^−1^ with a resolution of 2 cm^−1^. X-ray diffraction (XRD) analysis was performed to analyze the crystalline structures using a diffractometer (PAN analytical model X’PERT-PRO, Malvern, UK) with K_α_ radiation of 1.5418 Å and a scan rate of 10^o^/min. To evaluate the surface morphology, Field Emission Scanning Electron Microscope (FESEM) analysis was done using Nova Nano SEM 650, Hitachi, Tokyo, Japan. EDS microanalysis system (EDAX) was used to analyze the elemental constituents. For SEM, the sample surfaces were sputter-coated with gold. The surface area and pore size of the as-synthesized adsorbents were analyzed using Brunauer- Emmett-Teller (BET) analysis using an ASAP-2020 Micrometrics surface area and porosity analyzer, USA. The interaction between the metal and the adsorbents were analyzed using X-ray photoelectron spectroscopy using an AXIS Ultra DLD XPS. The boron analysis was carried out using an inductively coupled plasma– optical emission spectrometry (ICP-OES) (Thermo Scientific—iCAP 6300—ICP-OES CID Spectrometer). All samples were analyzed and stored in plastic bottles to avoid any contamination from borosilicate glass.

### Batch adsorption experiments

Batch adsorption experimental procedure was followed to obtain information about the boron equilibrium in the system and to study the effect of variable parameters such as adsorption time, pH, initial concentration, and temperature. A stock solution of 100 ppm boron (source of boron was B(OH)_3_) was prepared in a 1L volumetric flask with deionized water.

#### The effect of pH on adsorption

The adsorption experiments were carried out at different pH values, which cover the acidic and basic conditions, i.e., pH 2, 4, 6, 8, and 10. Initially, 50 mL of 100 ppm boron stock solution is taken in a plastic bottle. The pH of the solution is adjusted in the proposed range using NaOH (0.05 mol/L) or HCl (0.05 mol/L). The adsorbent dosage was 1 g/L. All samples were kept at constant shaking at 150 rpm for 24 h on a temperature controlled mechanical shaker at room temperature (25° C).

#### Effect of concentration on adsorption

After understanding the optimum pH for the adsorption for the adsorbents, the effect of initial concentrations was analyzed. Various concentrations of boric acid solutions were prepared (10, 20, 30, 40, 50, 60, 70, 80, 90, and 100 mg/L) and the effect of concentration was studied by keeping the amount of adsorbent and temperature constant. The amount of boron in the solution was calculated and was in the range (1.75–17.50) mg/L. To 50 mL of boron solution with various concentrations, 1 g/L adsorbent was added and the reaction was kept under constant shaking at 150 rpm for 24 h. at 25 °C. All experiments were done in triplicate.

#### Fitting of adsorption isotherms

To understand the mechanism of adsorption, the adsorption isotherms for the experiments were deduced from the results. Simple and widely used models were used in this study. The relation between equilibrium adsorption capacity and equilibrium concentrations were fitted to four isotherm models, namely Langmuir (Eq. 1) (Igwe & Abia, 2007), Freundlich (Eq. 2) (Chakraborty et al. 2016), Dubinin-Radushkevich (Eq. 3) (Chaudhry et al.^2017^), and Temkin (Eq. 4) (Du et al. 2011). Among these, the Langmuir model assumes that the adsorbent surface is homogenous and contains one type of binding site whereas the Freundlich model considers multilayer adsorption on a heterogeneous surface.

1 $${q}_{e =}\frac{{q}_{m}{K}_{L}{C}_{e}}{1+ {K}_{L}{C}_{e}}$$

2 $${q}_{e}={K}_{F}{C}_{e}^\frac{1}{n}$$

3 $${q}_{e}={q}_{D}\mathrm{exp}(-{K}_{D}{\varepsilon }^{2})$$

4 $${q}_{e }=B\mathrm{ln}\left({K}_{T}{C}_{e}\right)$$

where q_e_ (mg/g) is the equilibrium adsorption capacity per gram dry weight of the adsorbent, K_L_ is the Langmuir constant related to the free energy of adsorption (L/mg), C_e_ is the solute concentration at equilibrium (mg/ L), K_F_ and n are Freundlich constants which give a measure of adsorption capacity and adsorption intensity, ε is Polanyi potential, q_D_ is the adsorption capacity (mol/ g), K_D_ is the Dubinin–Radushkevich constant related to adsorption energy (mol^2^/ kJ), B = RT/b (R is the ideal gas constant (8.314 J/ K mol), T is the absolute temperature (K), b is the Temkin constant related to the heat of sorption (J/ mol), and K_T_ is the Temkin isotherm constant (L /g).

#### Effect of temperature on the adsorption

The effect of temperature on adsorption was analyzed at three different temperatures including 25 °C, 35 °C, and 45 °C. Different concentrations of the boric acid solutions were prepared (10 mg/L -100 mg/L, i.e., 1.75–17.50 mg/L boron) and analyzed by keeping the boric acid solution (50 mL) and adsorbent dosage (1 g/L) constant at pH 8. The concentration of boron in each boric acid solution was calculated and analyzed. The feasibility of the adsorption process can be understood from the thermodynamic calculations. The thermodynamic parameters were calculated from the following Eqs. 5 & 6 (Chakraborty et al. 2016):

5 $${\Delta G}^{0}=-RTln {K}_{L}$$

6 $${\Delta G}^{0}={\Delta H}^{0}-{T\Delta S}^{0}$$

where ΔG^o^ (standard Gibbs free energy change, kJ/mol), ΔH^o^ (standard enthalpy change, kJ/mol), ΔS^o^ (standard entropy change, J/mol. K), R – the gas constant (8.314 J/mol. K), T- the temperature in Kelvin (K) and K_L_ Langmuir equilibrium constant.

## Results and discussions

In this study, the adsorbents were fabricated from bio-waste-based materials namely *os sepiae,* and modified with several methods that might improve absorption capacity. Compared to other adsorbents reported, the collection and processing of *os sepiae* into various forms whether in particles or powder form is very simple, rapid, and cost-effective. Sample CFB was fabricated by a simple powdering of rough cuttlefish bone and subsequent sieving. The metal adsorption capacity of the adsorbents can be improved by various chemical treatments (Gendy et al. 2021; Mandal et al. 2021; Kurniawan et al. 2006), leading to suitable functionality and porosity, which may affect the absorption efficiency of metal cations (Lam et al. 2007). Thus, the CFB-D sample was prepared by treatment with NaOH solution, NaOH was chosen due to deprotonation effects and hence the surface charge on the adsorbent can be tailored by its reaction (Tanimoto et al. 2004). Moreover, the *os sepiae* contains mucus polysaccharides, proteins, and chitin in its structure which can be easily changed in the alkaline solution (Sophia & Sakthieswaran 2019). This will result in the exposure of more appropriate functional groups that can bind the metal ions on the surface of the adsorbent. Another modification of CFB leads to CFB-A sample by treatment in acidic conditions and subsequent reaction with EDTA. In such a fabrication process, it was assumed the formation of pores with acidic treatment and further a chelating complexation of Ca^2+^ ions and subsequent removal from the surface to create a binding site for other ions (Chen et al. 2010). Furthermore, adsorbed EDTA molecules can occupy the porous sites of the *os sepiae* structure improving metal chelate bonds (Babu et al. 2018).

### Characterization of adsorbents

#### X-ray diffraction analysis

**Fig. 1:**
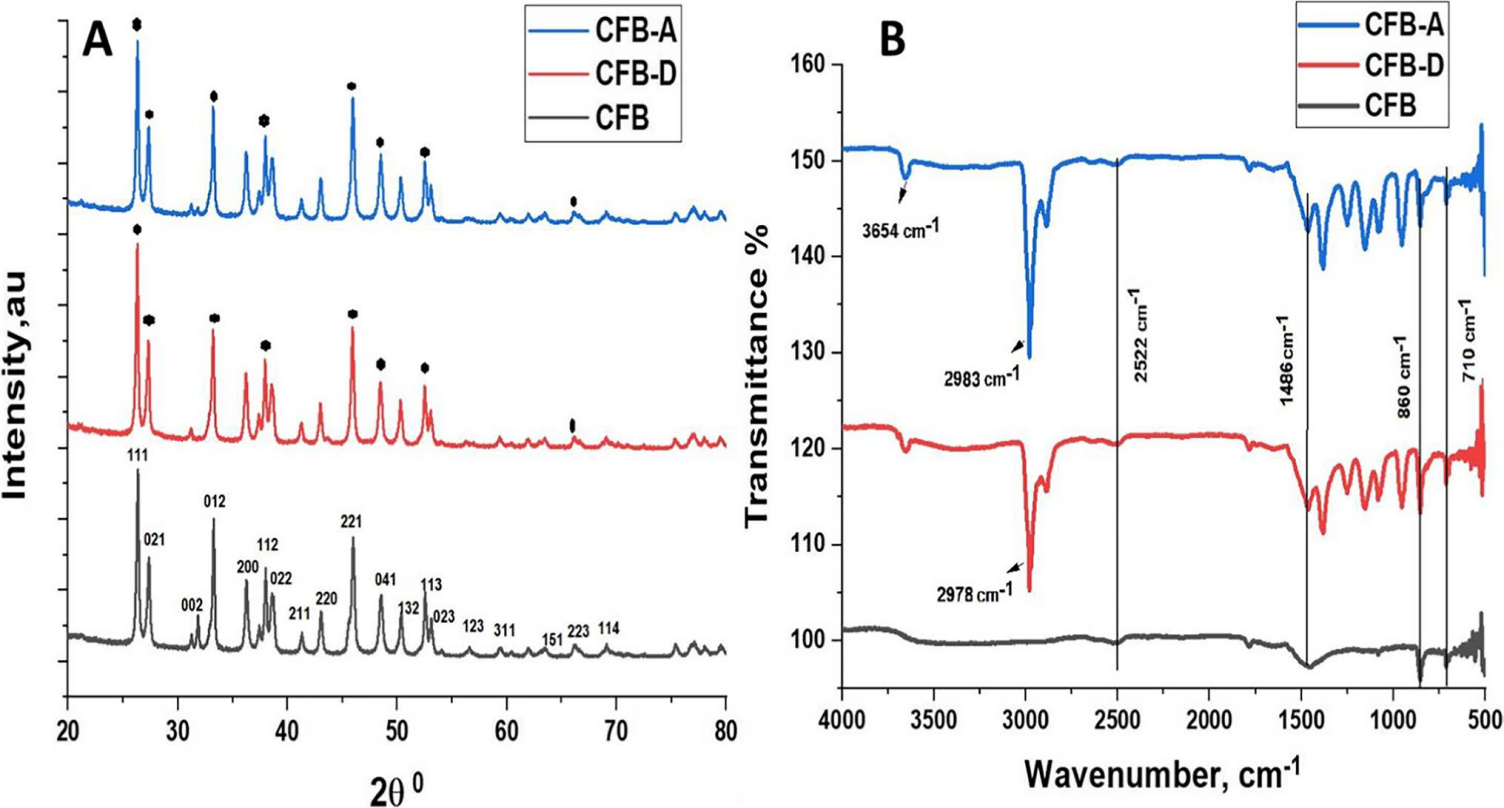
(**A**) X-ray diffraction spectra. (**B**) Fourier transform infrared spectra of CFB, CFB-D, and CFB-A

The surface properties and the morphologies of the adsorbents together with their effect on the adsorption are evaluated. Figure 1(A) represents the X-ray diffraction pattern of the CFB and its modified forms. The characteristic peaks of aragonite present with proper description in the bottom spectrum confirm the nature of the crystalline material and it matches with the crystal structure of orthorhombic aragonite (JCPDS file 75–2230). In the case of deprotonated cuttlefish bone (CFB-D), the crystalline nature of the powder remains the same however there is a slight increase in the intensity of (211) peak when compared to the CFB and CFB-A. The activated cuttlefish bone (CFB-A) shows no difference in the XRD structure when compared to CFB.

#### FTIR spectroscopy analysis

The functional groups present in the adsorbents were analyzed using the Fourier transform infrared spectra (FTIR) which are given in Fig. 1(B). The *os sepiae* have a large number of functional groups present on its surface (Jung et al. 2018). The absorption bands at 710 cm^−1^, 860 cm^−1^, 1080 cm^−1^, 1486 cm^−1^, and 2522 cm^−1^ for the CFB indicate the existence of aragonite carbonates (Balu et al. 2020). The functional groups including –CH = CH- at 710 cm^−1^, CH_2_ = C < RR^’^ at 860 cm^−1^, -C-NH_2_ at 1080 cm^−1^, -NH_3_^+^X^−^ at 2522 cm^−1^ etc. are present. The sharp peak at 710 cm^−1^ can be assigned to Ca-O bonds (Witoon 2011). A small peak at 1486 cm^−1^ can be related to C = O vibrations (Ahmad et al. 2012). In the case of the CFB-D sample and different functional groups present on the surface of the CFB are exposed. A small broad peak appearing at 3657 cm^−1^ indicates the presence of -O–H stretching vibrations (Aroke et al. 2013). New peaks were observed confirming modification such as a sharp peak at 2984 cm^−1^ indicating the asymmetric stretching of the presence of –CH_3_ from amide group (Florek et al. 2009), 1357 cm^−1^, 1253 cm^−1^, two groups of –C-NH_2_ at 1146 cm^−1^ and 952 cm^−1^ appeared after the treatment. CFB-A sample also showed new peaks with slight shifts from that of CFB-D. CFB-A also exhibited a small broad peak at 3654 cm^−1^ attributed to the –O–H and –N–H stretching vibration.

#### Energy-dispersive X-ray spectroscopy (EDX) analysis

The EDX analysis was done to understand the constitution of the CFB and its modified forms and the results are given in Table 1. CFB which is rich in CaCO_3_ also has some trace amount of proteins (3–7%) and chitin (3–4%) in it (Chakraborty et al. 2016). In the CFB-D sample, the mass % of calcium slightly decreases due to the etching of metal from the surface owing to deprotonation. In the case of CFB-A, there is a 48% increase in the mass % of N than that of CFB which indicated the exposure of amide groups and that some of the EDTA might have remained in the pores of the adsorbent through physical interaction.

**Table 1:** EDX results of CFB, CFB-D, and CFB-A

Element	Mass %		
	CFB	CFB-D	CFB-A
C	15.99	16.54	18.66
O	41.65	41.11	40.71
N	8.06	9.62	16.03
Ca	34.31	32.72	24.59
Total	100.00	100.00	100.00

#### BET analysis

The surface area and pore structure of the adsorbents were analyzed using BET adsorption–desorption isotherm, which corresponded to a type IV isotherm, indicating the presence of a mesoporous structure. Table 2 represents the BET adsorption parameters of the adsorbents. The average pore diameter of the CFB, CFB-D, and CFB-A is found to be 6.8 nm, 15.1 nm, and 21.8 nm, respectively, confirming the existence of a mesoporous structure with a surface area of 3.4 m^2^/g, 11.8 m^2^/g, and 39.8 m^2^/g, respectively. The increase in surface area of the treated CFB-D and CFB-A is helpful for the adsorbate molecules approach to the adsorbent active sites, which can enhance the adsorption efficiency.

**Table 2:** Surface area and pore size of CFB, CFB-D, and CFB-A

Adsorbent	Particle size (µm)	Pore size (nm)	Pore Volume (cm^3^/g)	Surface area (m^2^/g)
CFB	17.6	6.8	0.021	3.4
CFB-D	5.1	15.1	0.027	11.8
CFB-A	1.5	21.8	0.059	39.8

### Boron adsorption study

#### Effect of pH on boron removal

The effect of pH on the boron adsorption efficiency is highly significant considering the fact that it affects the complexation reactions between the metals and the adsorbents.

**Fig. 2:**
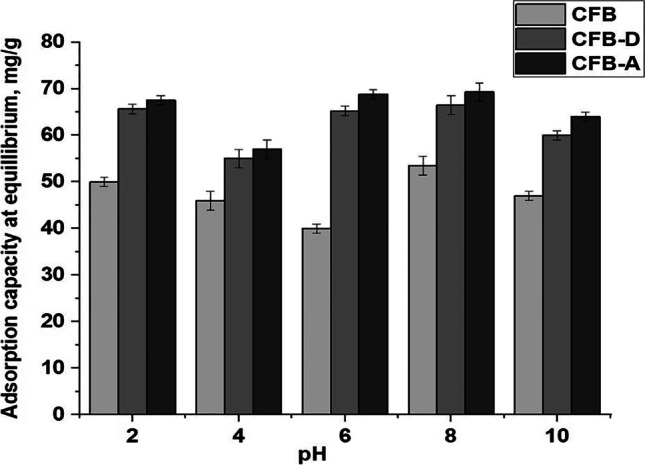
Effect of different pH values on the removal of boron by CFB, CFB-D and CFB-A. (Experimental conditions: Initial boron concentration: 100 mg/L (50 mL), Adsorbent mass: 0.05 g, Agitation speed: 150 rpm, contact time: 24 h, Temperature: 25 °C, pH: 2–10)

At different pH values, the electrostatic attraction between the adsorbent and the boron varies due to the variation in its structural and electronic properties. Figure 2 represents the effect of pH on the boron adsorption using CFB, CFB-D, and CFB-A samples. It can be seen that the removal of boron by the adsorbents varies based on applied pH. In the case of CFB, the maximum adsorption capacity of 53.8 ± 0.04 mg/g was shown at pH 8. After modification, CFB-D has an adsorption capacity of 66.45 ± 0.02 mg/g at pH 8 and CFB-A shows the maximum adsorption capacity at pH 8 which is 69.8 ± 0.02 mg/g. It is also interesting to see that compared to CFB, the modified CFB-D and CFB-A have good adsorption capacity over a wide range of pH.

To understand the surface charge on the adsorbents, the pH at the point of zero charges (pHpzc) was measured. The results showed pHpzc values of 7.1, 6.5, and 7.6 for CFB, CFB-D, and CFB-A respectively. All three adsorbents showed near-neutral character on their surface. These pHpzc values suggest the possibility that the surface charge predominance of the adsorbents can depend on the pH of the solution. Furthermore, it can have a predominance of positive charges when the pHsolution > pHpzc while for pHsolution < pHpzc the surface tends to be predominantly negative.

The adsorption capacity of adsorbent on a particular pH depends on different factors including the structure of the adsorbent, the charge on its surface, and electrostatic interaction between the adsorbent and the metal ion. pH can both alter the site dissociation on the surface of the adsorbents and also the solution chemistry of the metal solution (Wang and Chen 2006). Dissolved boron mainly exists in the form of boric acid or borate ions at low concentrations (≤ 216 ml/L). Hence the boron species present in the current study can be assigned mainly as mononuclear borate,${(B(OH)}_4^-)\;and\;\mathrm B{(\mathrm{OH})}_3$. The main component of *os sepiae* is calcium carbonate; there exists a possibility of releasing ions such as HCO_3_^−^, CO_2_^−^ and OH^−^ at higher pH, which in turn results in the possibility of precipitation of metals in their hydroxide forms an electrostatic repulsion (Abdel-Khalek et al. 2017). The increase in adsorption capacity at lower pH for boron can be linked to the protonation or the increased concentration of hydronium cation [H_3_O^+^] on the surface groups of the adsorbent (Wang and Chen 2006). This electrostatic attraction might facilitate the adsorption of borate ions on the CFB surface. As the pH increases, the presence of negatively charged sites on the adsorbent increases thereby creating the possibility of electrostatic repulsion between the adsorbent surface and the boron.

As the surface of the CFB-D and CFB-A contains plenty of functional groups, the affinity of the adsorbents towards various boron species improves and there is a possibility for complexation reaction of B(OH)_3_ and (${B\left(OH\right)}_{4}^{-}$ with the OH^−^ group on the adsorbent surface. The pH also has a direct effect on the solution chemistry of the metals. It is well-known that at higher pH, the concentration of borate ion (${B\left(OH\right)}_{4}^{-}$ anion) is higher than that of other boron species (Schott et al. 2014). These borate anions can favorably interact with amino and hydroxyl groups. The optimal pH for the removal of boron varies with adsorbents. For example, the optimal pH to remove boron using adsorbent derived from waste tire rubber is 2 (Babiker et al. 2019) whereas adsorbent derived from eggshell waste showed maximum efficiency for boron removal at pH 8 (Al-Ghouti and Khan 2018). In this study, both CFB-D and the activated CFB-A have shown improved adsorption capacity for all investigated pH.

#### Effect of initial boron concentration

**Fig. 3:**
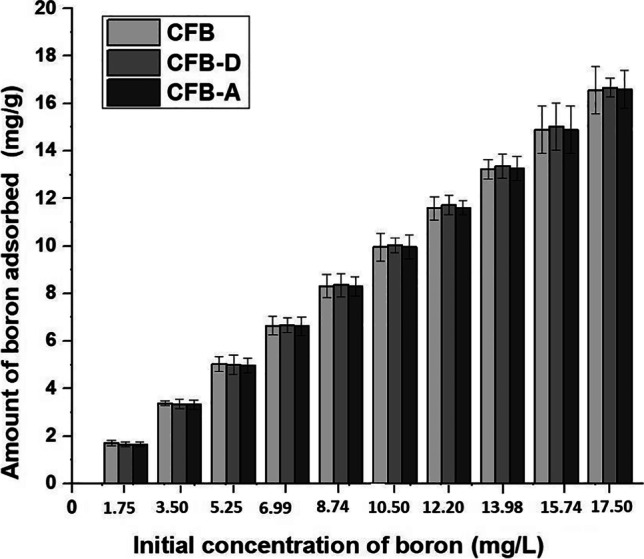
Effect of initial concentrations on boron adsorption onto CFB, CFB-D and CFB-A. (Experimental conditions: Initial boron concentration: 1.75 mg/L – 17.50 mg/L (50 mL), Adsorbent mass: 0.05 g, Agitation speed: 150 rpm, contact time: 24 h, Temperature: 25 °C, pH: 8)

The effect of initial boron concentration on the efficiency of boron adsorption onto CFB, CFB-A, and CFB-D is shown in Fig. 3. It is evident that the boron adsorption onto the CFB, CFB-D, and CFB-A increased as the initial concentration increased, which follow a positive progression. For CFB and CFB-D, the adsorption is linear and increases slowly by the increase in concentration. This is probably due to the initial availability of a vast number of pores as adsorption sites and supporting functional groups present on the adsorbent. Furthermore, after 7.5 mg/L, a constant and steady behavior is shown by all three adsorbents. This may be due to the decreased availability of pores and active sites in the adsorbent. In the case of CFB-A, even though there is an increase in boron adsorption with respect to initial concentration, there is a decreasing trend for the efficiency of the boron removal up to 6.99 mg/L followed by a constant trend in the adsorption. In comparison, alkali-treated CFB-D is showing effective adsorption of boron throughout the concentrations studied.

#### Effect of temperature

**Fig. 4:**
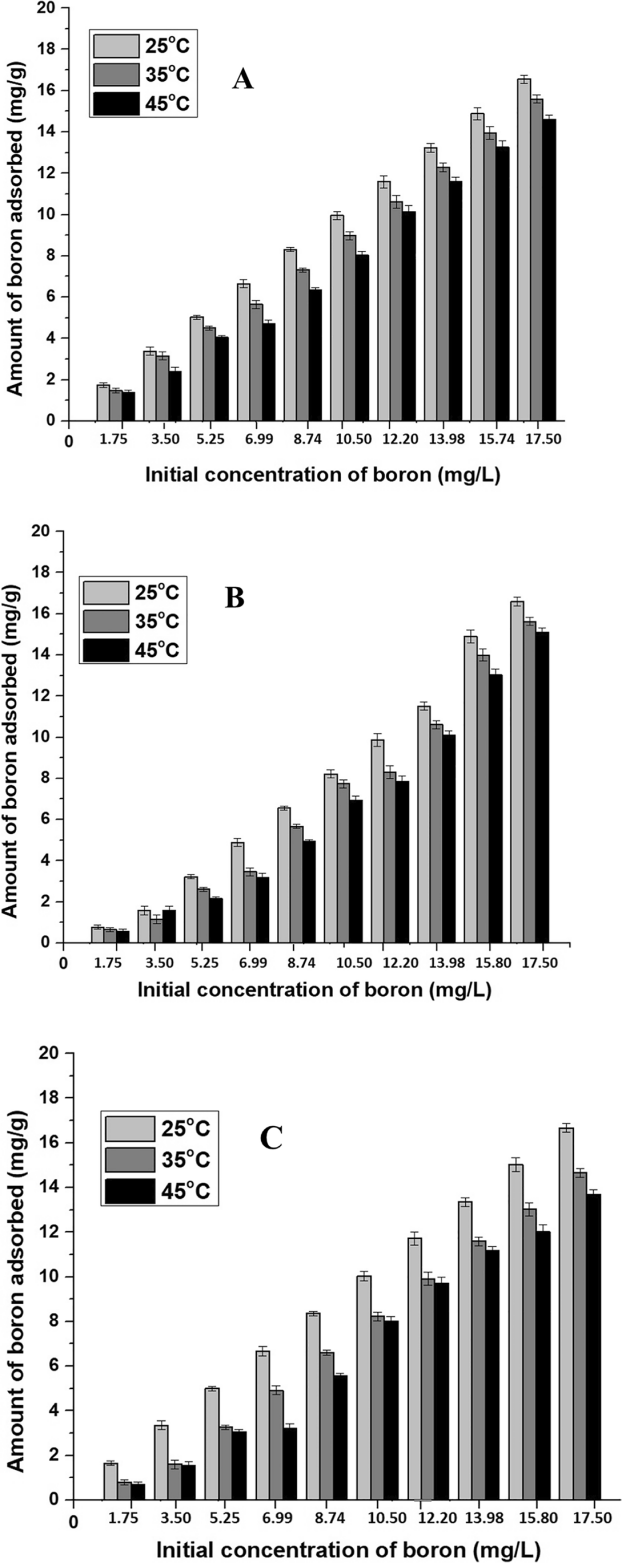
Effect of temperature on boron adsorption onto (**A**) CFB (**B**) CFB-D and (**C**) CFB-A. (Experimental conditions: Initial boron concentration: 1.75–17.50 mg/L (50 mL), Adsorbent mass: 0.05 g, Agitation speed: 150 rpm, contact time: 24 h, Temperature: 25 °C, 35 °C & 45 °C, pH: 8)

The role of temperature in the adsorption process is highly significant considering the fact that it can affect the active binding sites in the adsorbents. Metal adsorption reactions are generally classified in the exothermic reactions category. The effects of temperatures on boron adsorption onto the CFB, CFB-D, and CFB-A in the range 25–45° C were investigated and are shown in Fig. 4. Figure 4A which represents the CFB, showed a linear increasing tendency with the removal of boron through adsorption when the initial concentration was increased. However, the percentage of adsorption was maximum for the adsorption at 25 °C. In the case of CFB-D (Fig. 4B) at initial concentrations, low temperature is favorable. As the concentration of boron increases, high temperature, i.e., 35 °C is giving high percentage removal of boron. CFB-D and CFB-A generally showed a linear increase in the adsorption capability with respect to initial concentration at various temperatures. For all the three adsorbents studied, at a higher temperature like 45 °C, the adsorption capacity decreases. This might be due to the increase in kinetic energy of the boron ions in the solution and also due to the exothermic nature of the adsorption process. However, the percentage removal of boron by CFB, CFB-D, and CFB-A is not drastically affected by the range of temperatures in this study. Careful analysis reveals that in the case of CFB, CFB-D, and CFB-A, the highest removal percentage of boron was observed at 25 °C, 35 °C, and 25 ^o^C respectively. Similar results were reported in the case of adsorption of Cu by cuttlefish bone powder where the temperatures up to 45 °C do not have any significant influence on the adsorption capacity of the adsorbent (Li et al. 2010).

**Fig. 5:**
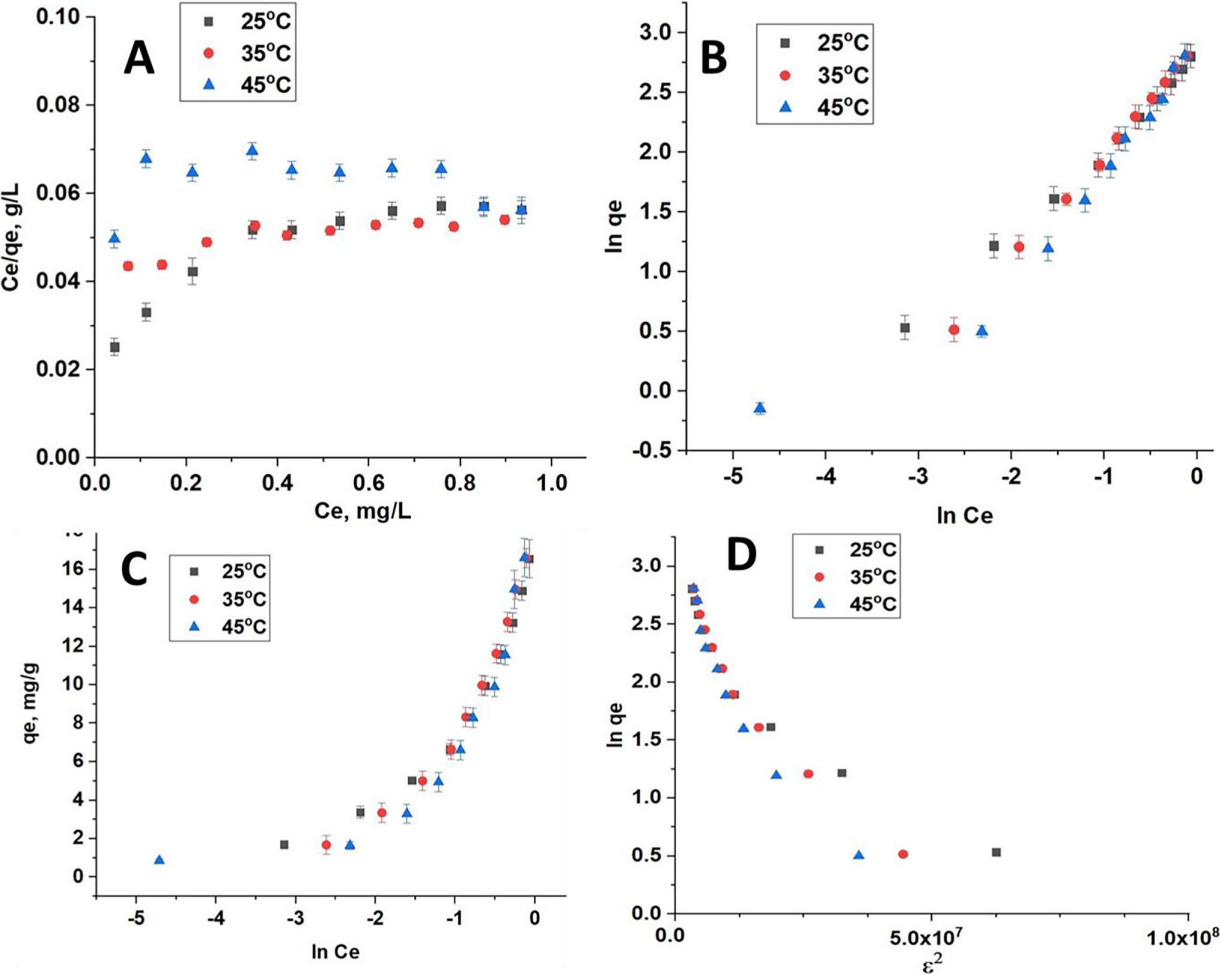
The linear adsorption isotherms for (**A**) Langmuir, (**B**) Freundlich, (**C**) Temkin, (**D**) Dubinin-Radushkevich at 25.0 °C, 35.0 °C, and 45.0 °C for CFB

**Fig. 6:**
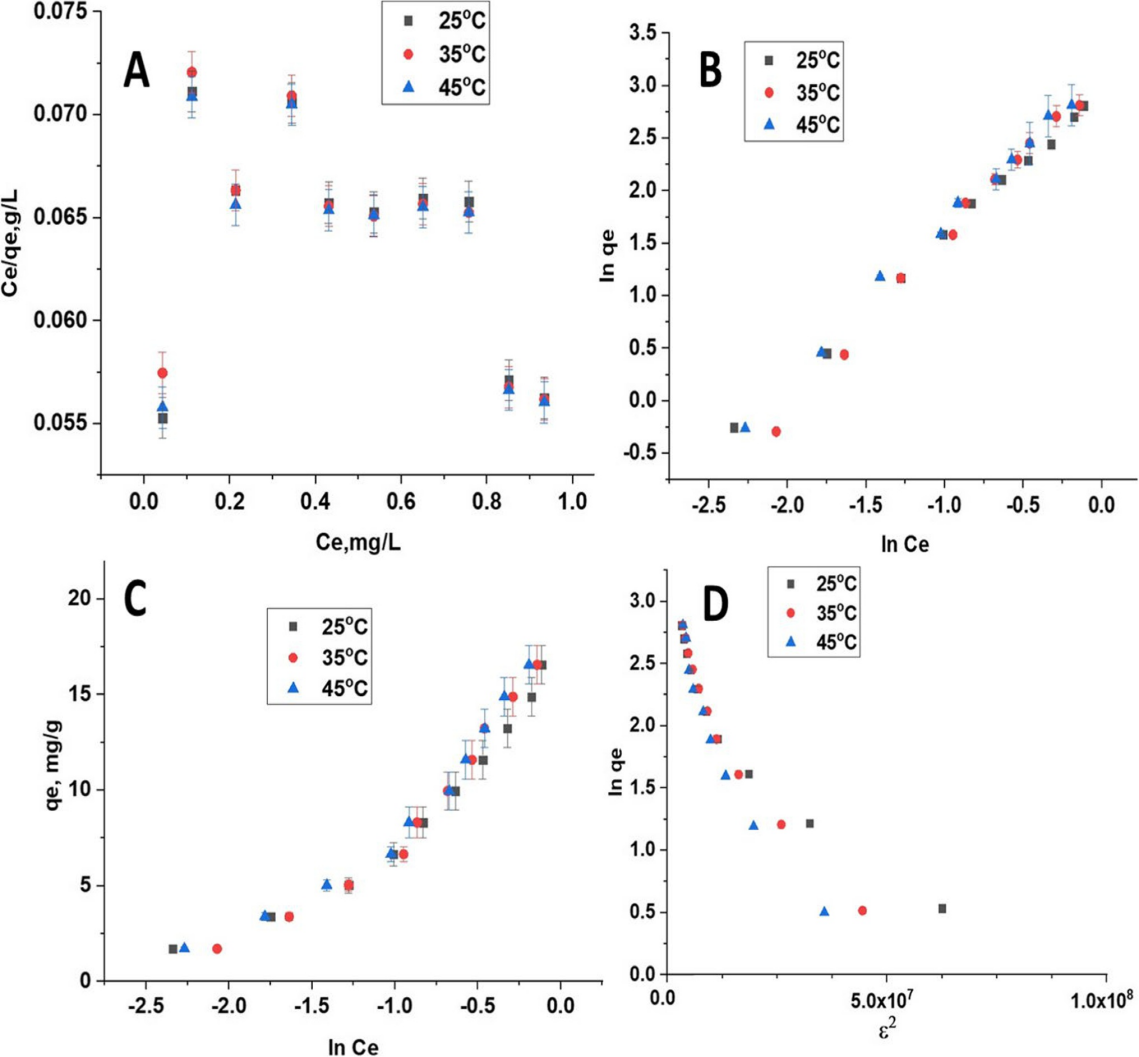
The linear adsorption Isotherms for (**A**) Langmuir, (**B**) Freundlich, (**C**) Temkin, (**D**) Dubinin-Radushkevich at 25.0 °C, 35.0 °C, and 45.0 °C for CFB-D

**Fig. 7:**
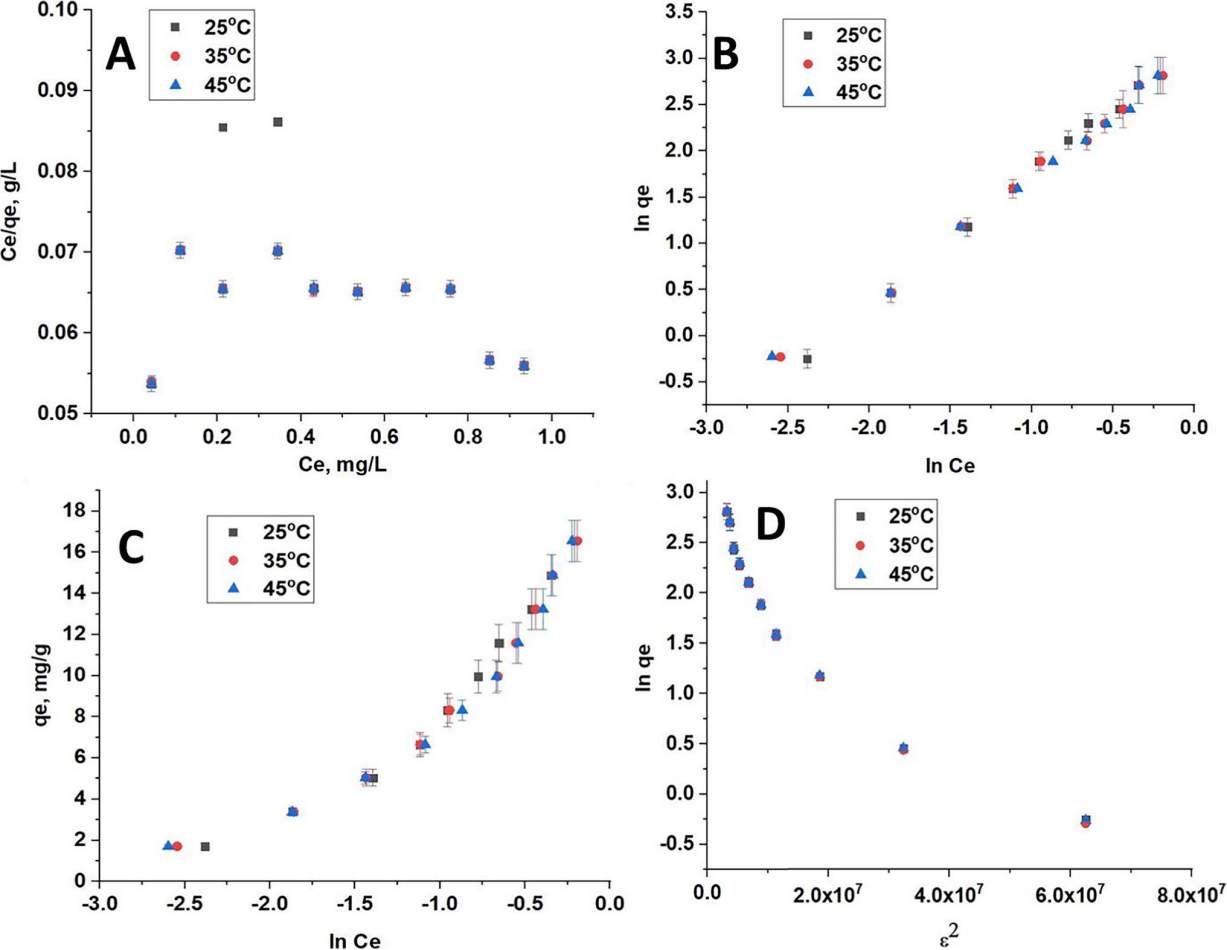
The linear adsorption Isotherms for (**A**) Langmuir, (**B**) Freundlich, (**C**) Temkin, (**D**) Dubinin-Radushkevich at 25.0 °C, 35.0 °C, and 45.0 °C for CFB-A

The predictive modeling procedure for the analysis and design of an adsorption process can be carried out using assistance from various adsorption isotherms. In this study, four adsorption isotherm models were applied; Langmuir, Freundlich, Temkin, and Dubinin-Radushkevich to describe the adsorption phenomena and estimate energy parameters. Figures 5, 6, and 7 show the fitted linear adsorption isotherms of CFB, CFB-D, and CFB-A respectively for all the studied models. The values of the isotherm parameters are given in Table 3. From the detailed analysis of the correlation coefficient obtained for different models, it can be concluded that the Freundlich model gives a good fit to the experimental data which also indicates the heterogenic nature of the adsorbent. From Langmuir adsorption isotherm, the K_L_ value, which indicates the favorability of the boron adsorption, is positive in the case of CFB, CFB-D, and CFB-A confirming the adsorption of boron to the CFB surface is energetically feasible (Chaudhry et al. 2017). In the case of CFB-D and CFB-A, the K_L_ value was maximum at 35 °C. From the Freundlich adsorption isotherm values, the K_F_ values were found to be increasing while increasing the temperature to 35 °C and then decreasing at 45 °C. The increase in K_F_ value might be because the temperature is improving the contact between the adsorbate and the adsorbent site. It is also to be noted that the K_F_ value of CFB-A is higher than that of other counterparts indicating that CFB-A has more affinity towards boron than others (Daraei et al. 2015). However, further increase in temperature is decreasing the K_F_ value which supports the earlier findings that an increase in temperature decreases the adsorption efficiency for an exothermic reaction. The correlation coefficient (R^2^) which is in the range 0.994–0.998 suggests that this model can be used to explain the experimental data. The heterogeneous and favorable nature of the system is revealed by the n value, which is greater than 1 for all systems. Freundlich isotherm assumes that the uptake of boron ions happens on a heterogeneous surface with a non-uniform distribution of heat of adsorption over the surface. It can also be inferred that there is a possibility for multilayer adsorption of boron on the adsorbent surface. The decrease in K_F_ value at 45 °C indicated that the adsorption efficiency decreases with an increase in temperature, which supports earlier findings.

**Table 3:** Isotherm parameters for CFB, CFB-D, and CFB-A

Langmuir model				Freundlich model			
Sample	Q(mg/g)	K_L_(L/mg)	***R***^**2**^		K_F_(mg/g)	***n***	***R***^**2**^
CFB				CFB			
25 °C	31.43	0.963	0.772	25 °C	3.09	1.38(2.00)	0.994
35 °C	84.10	0.265	0.743	35 °C	18.17	1.10	0.998
45 °C	10.10	0.278	0.662	45 °C	14.47	1.99	0.982
CFB-D				CFB-D			
25 °C	156.03	0.095	0.122	25 °C	19.17	1.07	0.998
35 °C	119.23	0.123	0.116	35 °C	24.04	1.11	0.994
45 °C	127.12	0.101	0.144	45 °C	23.49	1.18	0.995
CFB-A				CFB-A			
25 °C	130.02	0.075	0.083	25 °C	24.15	1.29	0.997
35 °C	111.34	0.083	0.089	35 °C	21.33	1.36	0.994
45 °C	85.24	0.075	0.083	45 °C	19.73	1.23	0.997
Temkin model				Dubinin-Radushkevich model			
CFB	K_T_(L/g)	B (J/mol)	**R**^**2**^	CFB	Q_D_(mg/g)	K_D_ (mol^2^ /kJ^2^)	R^2^
25 °C	20.08	4.65	0.864	25 °C	13.42	3.66 × 10^–8^	0.886
35 °C	12.34	5.92	0.886	35 °C	15.50	5.46 × 10^–8^	0.929
45 °C	43.96	3.14	0.595	45 °C	9.19	1.98 × 10^–8^	0.653
CFB-D				CFB-D			
25 °C	9.68	6.62	0.906	25 °C	12.26	5.02 × 10^–8^	0.886
35 °C	7.79	7.86	0.927	35 °C	12.37	5.09 × 10^–8^	0.929
45 °C	9.45	7.12	0.913	45 °C	12.36	5.04 × 10^–8^	0.653
CFB-A				CFB-A			
25 °C	16.14	6.90	0.927	25 °C	12.38	5.02 × 10^–8^	0.871
35 °C	11.59	6.31	0.889	35 °C	12.32	4.99 × 10^–8^	0.868
45 °C	12.07	6.13	0.869	45 °C	12.29	4.98 × 10^–8^	0.867

The Temkin isotherm correlates the adsorption heat of molecules and the adsorbent surface and the distribution of binding energy. The Temkin adsorption constant, K_T_ was lowest in the case of CFB, CFB-D, and CFB-A at 35 °C compared to other temperatures studied. The heat adsorption constant, B also showed a decreasing tendency as the temperature increases except in the case of CFB-D. The correlation coefficients for the Temkin and Dubinin-Radushkevich models range between 0.595–0.864 and 0.653–0.929 respectively, which indicates that both models are not suitable to fit the adsorption curve for this experiment. Hence, to the level of the adsorbate concentration used in this study, the Freundlich isotherm model is the best fit. However, if the experiment is conducted at a higher adsorbate concentration, the data might fit well using the Langmuir isotherm (Chen et.al 2016). Table 4 shows a comparison of boron adsorption capacities with various adsorbents.

**Table 4:** Comparison of boron adsorption capacities with various adsorbents

Adsorbent	Adsorption Capacity (mg/g) at 25 °C	pH	Reference
CFB-A	69.80	8.00	This work
CFB-D	66.40	8.00	This work
CFB	53.80	8.00	This work
Eggshell membrane	42.19	6.00	Al-Ghouti and Khan 2018
Chemically modified eggshell membrane	31.06	6.00	Al-Ghouti and Khan 2018
Alginate gel beads	9.86	11.00	Demey-Cedeño et al.^2014^
Zeolite	0.03	10.00	Yüksel & Yürüm, 2010
Salicylic acid treated activated carbon	1.78	4.68	Cana et al. 2012
Fly ash	0.30	10.00	Yüksel & Yürüm, 2010
Waste tire rubber	5.32	2.00	Babiker et al. 2019
Chemically modified waste tire rubber	8.41	2.00	Babiker et al. 2019

The thermodynamic parameters for the adsorption process calculated from the lnK_d_ (Distribution constant, Kd = qe/Ce) versus 1/T linear plot for the CFB, CFB-D and CFB-A are given in Table 5. The negative value for the ΔG^o^ indicated that the adsorption reaction is spontaneous and feasible. An increase in the ΔG^o^ value with a rise in temperature indicates that the reactions favor low temperatures and are more feasible while keeping the temperature low. Furthermore, the exothermic nature of the adsorption process was confirmed by the negative values for enthalpy change (ΔH^o^). ΔS^o^, which shows the change in entropy, was found to be positive indicating that the solid–liquid interface increases randomly during the adsorption. It is to be noted that the capacity of adsorbents to remove pollutants from water depends on various factors including the nature of the adsorbent, pH of the solution, flow rate of the solution, the mode of test used (batch/fixed-bed column), bed height and so on (Stavrinou et al. 2020). Hence the boron removal efficiency and the thermodynamic parameters reported are based on the selective condition used in this study.

**Table 5:** Thermodynamic parameters for boron adsorption onto CFB, CFB-D, and CFB-A

Temperature °C	ΔG° (kJ/mol)	ΔH° (kJ/mol)	ΔS° (J/mol.K)
CFB			
25 °C	-34.10	-307	3.90
35 °C	-33.95		
45 °C	-33.84		
CFB-D			
25 °C	-58.31	-379	4.19
35 °C	-53.66		
45 °C	-60.48		
CFB-A			
25 °C	-63.94	-310	4.00
35 °C	-63.51		
45 °C	-68.27		

### Adsorption mechanisms

The mechanisms involved between the adsorbent and the boron species were investigated in detail using various spectral and microscopic methods.

#### FTIR analysis

**Fig. 8:**
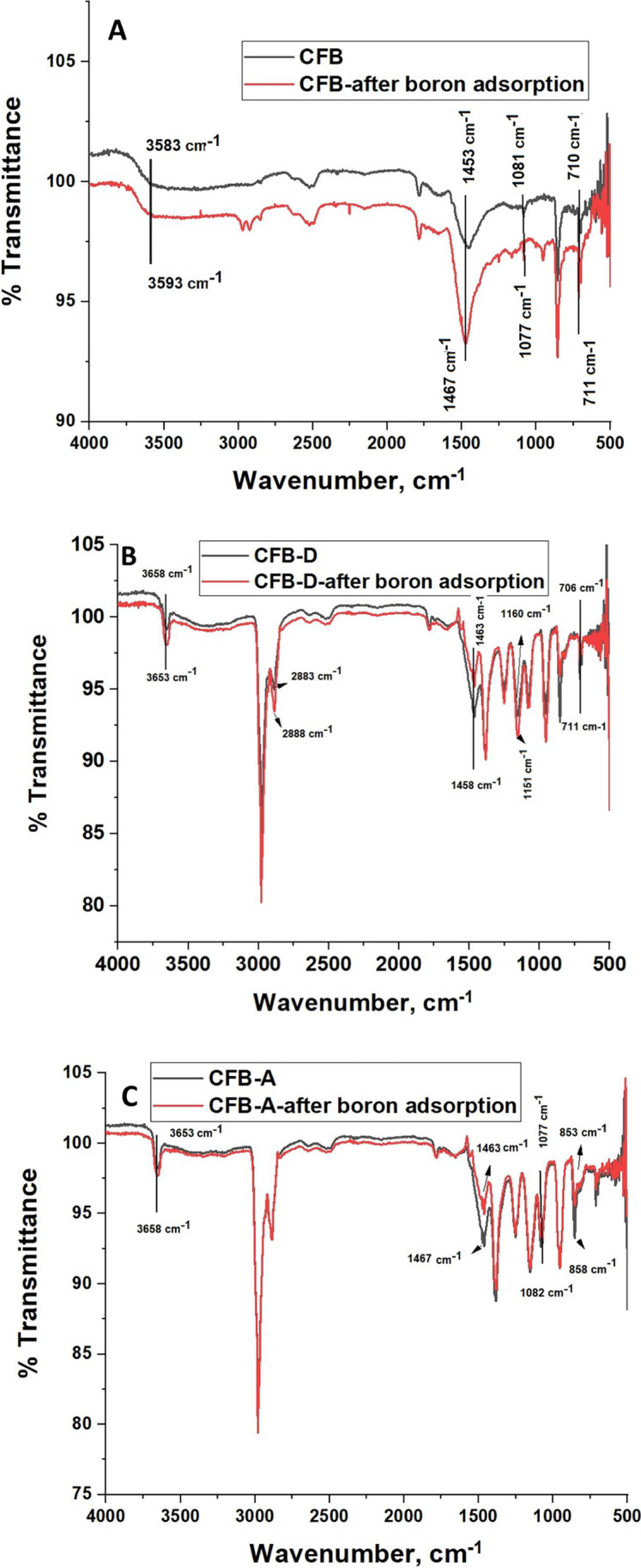
Fourier transform infrared spectra of (A) CFB, CFB-D and CFB-A, (B, C, D) before (black line) and after boron adsorption (red line) of CFB, CFB-A, and CFB-D respectively

The FTIR spectrum of the CFB, CFB-D and CFB-A after boron adsorption is given in Fig. 8. In the case of CFB given in Fig. 8A, the FTIR peak of > N–CH_3_ shifted from 1453 to 1467 cm^−1^ and –C–NH_2_ shifted from 1081 to 1077 cm^−1^ indicating the involvement of the amino group in bonding with borate. The broad peak at 3583 cm^−1^ shifted to 3593 cm^−1^, which indicates the presence of interaction between the hydroxyl group and the boron species. The characteristic –C-O stretching band for the aragonite plane structure at 710 cm^−1^ was also shifted to 711 cm^−1^. A similar trend is visible in the case of CFB-D and CFB-A (Fig. 8(B&C)).

**Fig. 9:**
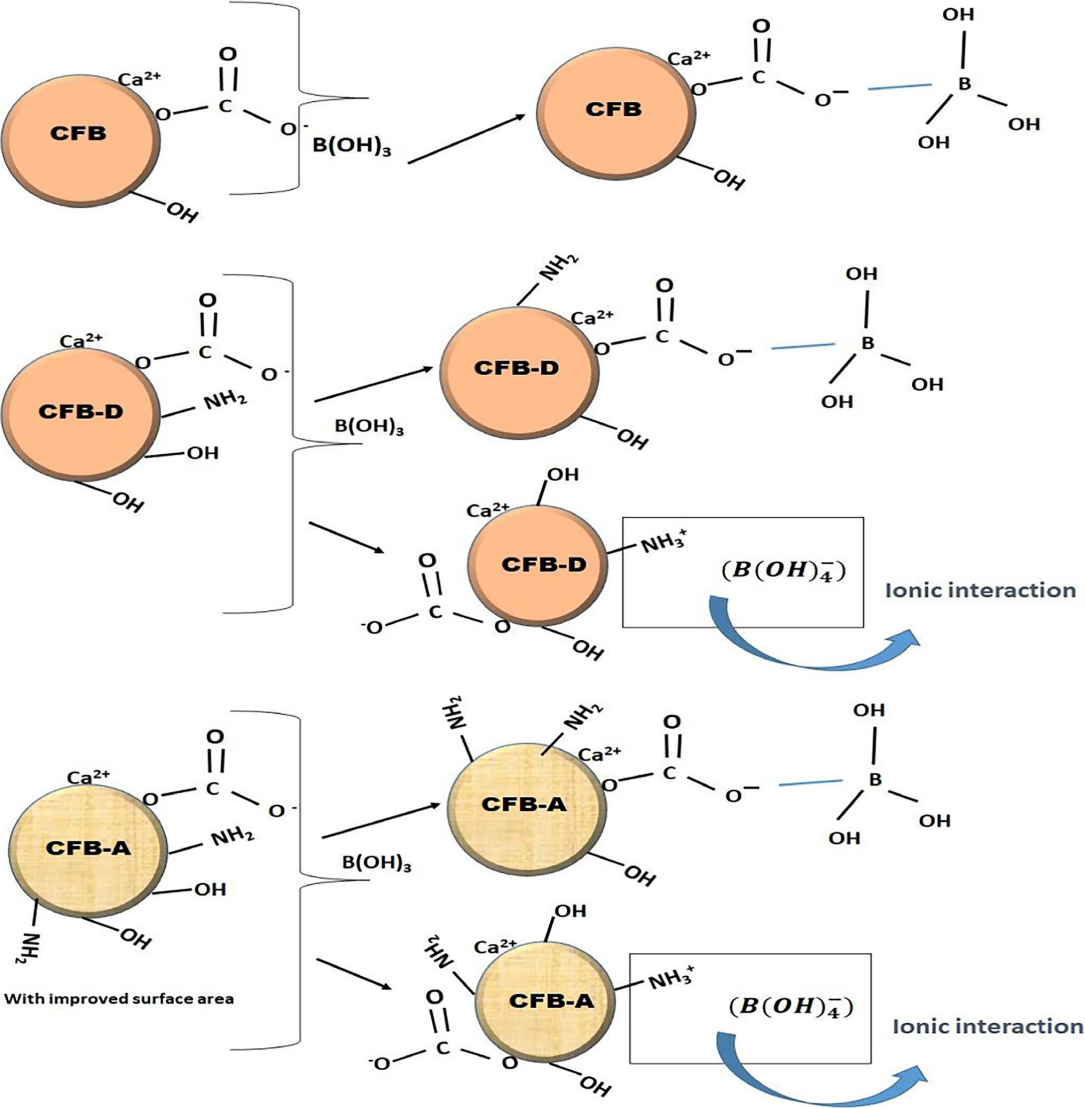
Schematic representation of possible interactions of CFB (upper), CFB-D (middle), and CFB-A (bottom) with boron

In CFB-D, similar to CFB, the peak at 1458 cm^−1^ shifted to 1463 cm^−1^, 1160 cm^−1^ shifted to 1151 cm^−1^ indicating the involvement of amide groups. The –C-O stretching band shifts from 711 cm^−1^ to 706 cm^−1^. The peak at 2883 cm^−1^ due to the asymmetric stretching of –CH_3_ was shifted to 2888 cm^−1^ after boron adsorption. In CFB-A after adsorption (Fig. 8(C)), the FTIR peak of > N–CH_3_ shifted from 1467 to 1463 cm^−1^, and the –C–NH_2_ peak at 1081 cm^−1^ was shifted to 1077 cm^−1^. In the case of CFB, CFB-D, and CFB-A, the peak at 853 cm^−1^ which is due to the internal vibration ${CO}_{3}^{2-}$ was shifted to 863 cm^−1^ indicating boron adsorption. This type of interaction was reported by Wang et al. (2013) when analyzing the adsorption of mercury on buckwheat husk. The analysis of the FTIR spectrum indicates the involvement of amino, carbonate (CO_3_)^2−^ and hydroxyl groups in the adsorption mechanism of boron. The possible mechanism of various interactions on the surface of adsorbents by boron is represented in Fig. 9.

#### Scanning electron microscopy analysis

**Fig. 10:**
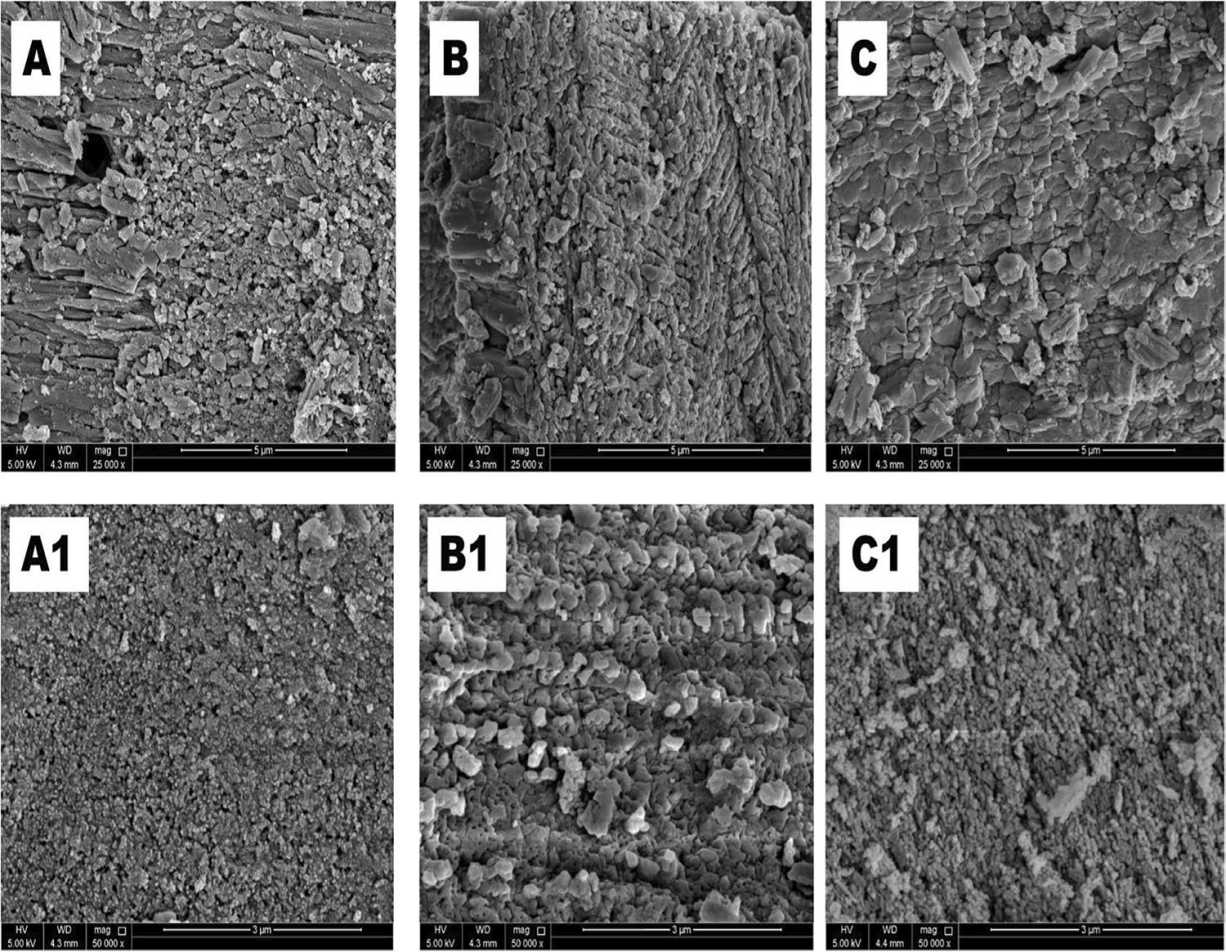
SEM images of (**A**) CFB (**B**) CFB-D and (**C**) CFB-A before boron adsorption and. (A1) CFB (B1) CFB-D and (C1) CFB-A after boron adsorption at 25 °C

The surface textural and morphological properties of CFB, CFB-D, and CFB-A were investigated using the field emission scanning electron microscope (FESEM). From the FESEM images shown in Fig. 10, it can be seen that there exists a lamellar-like structure for the CFB. After the chemical modification, the grain size of the CFB-A and CFB-D (Fig. 10B&C) seems to be decreasing which further enhances the porosity and effective surface area of the adsorbent (Sandesh et al. 2013). Figure 10(A1) represents the SEM image of CFB after boron adsorption clearly indicating that the porous and grainy nature of the CFB is retained after adsorption. Whereas in the case of CFB-D (Fig. 10B1), the grain size seems to be increased which indicates the possibility of agglomeration. This observation is similar to the report of Y.-Z li et al. which shows the alkali-treated *os sepiae* powder has changed its morphology to a rose-like crystal after adsorption of copper (Li et al. 2010). CFB-A (Fig. 10C1) also retains its porous structure after the adsorption process which indicates the stability of the adsorbent. The EDX analysis clearly shows the presence of boron after adsorption. It is to be noted that the percentage composition of boron is highest for the CFB-A sample that is in agreement with adsorption studies (Table 6).

**Table 6:** Energy-dispersive X-Ray spectroscopy results of CFB, CFB-D and CFB-A after adsorption

After boron adsorption					
CFB		CFB-D		CFB-A	
Element	Mass %	Element	Mass %	Element	Mass %
C	21.16	C	14.38	C	29.85
O	24.26	O	20.18	O	15.66
N	6.06	N	7.19	N	9.21
Ca	26.15	Ca	28.93	Ca	10.02
B	22.36	B	29.38	B	35.26
Total	100.00	Total	100.00	Total	100.00

#### XPS analysis

**Fig. 11:**
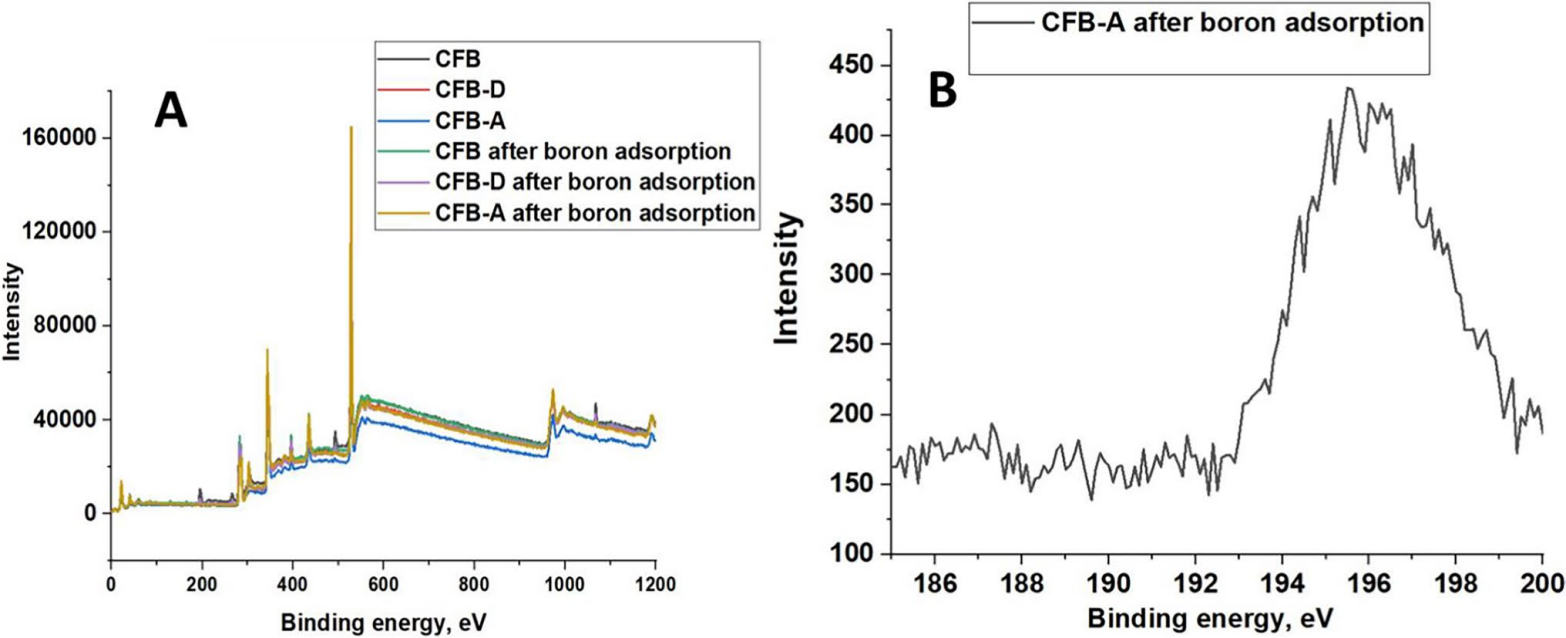
(**A**) High-resolution XPS spectra of CFB, CFB-D, and CFB-A before and after boron adsorption. (**B**) B1s spectra of boric acid after adsorption by CFB-A

**Fig. 12:**
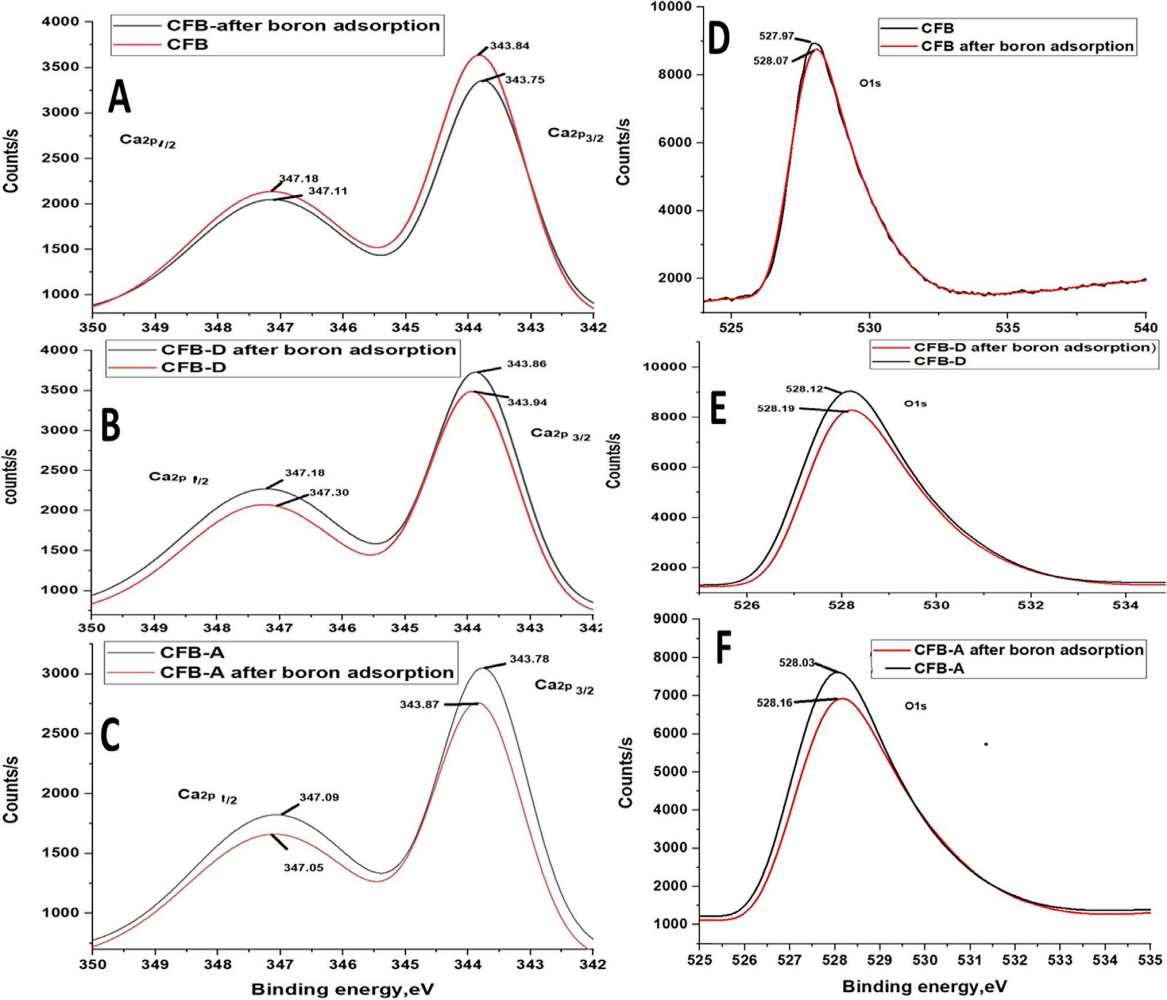
High-resolution XPS spectra of (**A**, **B**, &,**C**) Ca before and after adsorption (**D**, **E**, & **F**) O1s before and after adsorption of CFB, CFB-D and CFB-A respectively

X-ray photoelectron spectroscopic technique was also used to analyze the mechanism involved in the adsorption of boron by the adsorbents. The B1s peak in the case of all adsorbents after the adsorption reaction is evident from the fact that boron is being adsorbed on the substrate (Fig. 11(A)). Figure 11B represents the B1s spectra of boric acid after adsorption by CFB-A which has a binding energy of 95.5 eV. The binding energy of Ca 2p_3/2_, Ca 2p_1/2_ decreased after boron adsorption by the adsorbents (Fig. 12(A, B & C)) which indicates that there is some change in the electron density around the atom. The binding energy of O1s increased (Fig. 12(D, E & F)) which suggests that the Ca and oxygen would compensate with each other. The possible bonds formed in the adsorption process are B-O–H and Ca-O-B which again confirm the adsorption of boron onto the surface of the absorbent. It is inappropriate to infer that all the shift in energy change is due to boron adsorption. However, the shift indicates some coordination of the metals with the substrate and might be because of the complexation (Gomes et al. 2002). The mechanism of boron incorporation in calcites, which is a similar system was well explained by Wang et al., and they suggest only charged species like B(OH)_4_^−^ interact with the crystal surface in calcite (Wang et al. 2018).

The process of metal adsorption by bio-waste-based adsorbents largely depends on the surface functional groups like chitin, carbonyl, hydroxyl, and many atoms like N, O, and S. These groups can initiate adsorption mechanisms through different processes including absorption complexation, ion exchange, micro precipitation and many more. Shifts of IR stretching bands especially that of negatively charged groups like –NH_3_^+^X^−^ strongly suggest that the mechanism of adsorption in this system involves electrostatic adsorption and metal hydroxide condensation. Furthermore, the shifts in N-containing groups such as N-CH_3_, -NH and –C-NH_2_ imply the existence of a complexation mechanism followed by micro precipitation in the case of adsorption by *os sepiae*.

## Conclusions

The experimental results indicate that the os sepiae, which is biomass waste, can be successfully utilized for wastewater treatment for the removal of boron. The adsorbent studied is inexpensive and easy to process together with excellent biodegradability. The result obtained from batch experiments showed that the adsorption process in this is exothermic and pH-dependent and the optimal pH for higher-level boron uptake was found to be 8. The equilibrium data were well fitted with the Freundlich model with a correlation coefficient of 0.998. Acid-treated CFB-A was found to have higher removal capacity compared to alkali-treated CFB-D. The negative value of ΔG^o^ and the positive value ΔS^o^ showed that the adsorption of boron by cuttlebones was feasible and spontaneous. FTIR analysis confirms the role of various functional groups including calcium oxide, carbonyl and hydroxyl group on the surface of the CFB in the adsorption mechanism. XPS spectra further demonstrate the involvement of B-O–H and Ca-O-B bonds in the adsorption mechanism. The mechanism of adsorption is likely to include electrostatic adsorption, surface complexation, and surface deposition. Os sepiae, which is a bio-waste, is a promising adsorbent for boron removal considering its abundance, easy processability, and adsorptive capacity. Therefore, the ability of the biowaste, CFB to adsorb boron from desalinated water offers a promising technique for wastewater treatment.

## Data Availability

All available data related to the study is included in the manuscript.
